# Differential contributions of subthalamic beta rhythms and 1/f broadband activity to motor symptoms in Parkinson’s disease

**DOI:** 10.1038/s41531-018-0068-y

**Published:** 2018-11-05

**Authors:** Stephanie Martin, Iñaki Iturrate, Ricardo Chavarriaga, Robert Leeb, Aleksander Sobolewski, Andrew M. Li, Julien Zaldivar, Iulia Peciu-Florianu, Etienne Pralong, Mayte Castro-Jiménez, David Benninger, François Vingerhoets, Robert T. Knight, Jocelyne Bloch, José del R. Millán

**Affiliations:** 1 Defitech Chair in Brain-Machine Interface (CNBI), Center for Neuroprosthetics (CNP), Ecole Polytechnique Fédérale de Lausanne (EPFL), Lausanne, Switzerland; 2 Helen Wills Neuroscience Institute, University of California, Berkeley, CA USA; 3 Li Ka Shing Faculty of Medicine, University of Hong Kong, Hong Kong, P.R. China; 4 Department of Clinical Neurosciences (Neurology and Neurosurgery), University Hospital of Vaud (CHUV), Lausanne, Switzerland; 5 Service de Neurochirurgie, Hôpital de Sion, Hôpital du Valais, Valais, Switzerland; 6 Department of Psychology, University of California, Berkeley, CA USA

## Abstract

Excessive beta oscillatory activity in the subthalamic nucleus (STN) is linked to Parkinson’s Disease (PD) motor symptoms. However, previous works have been inconsistent regarding the functional role of beta activity in untreated Parkinsonian states, questioning such role. We hypothesized that this inconsistency is due to the influence of electrophysiological broadband activity —a neurophysiological indicator of synaptic excitation/inhibition ratio— that could confound measurements of beta activity in STN recordings. Here we propose a data-driven, automatic and individualized mathematical model that disentangles beta activity and 1/f broadband activity in the STN power spectrum, and investigate the link between these individual components and motor symptoms in thirteen Parkinsonian patients. We show, using both modeled and actual data, how beta oscillatory activity significantly correlates with motor symptoms (bradykinesia and rigidity) only when broadband activity is not considered in the biomarker estimations, providing solid evidence that oscillatory beta activity does correlate with motor symptoms in untreated PD states as well as the significant impact of broadband activity. These findings emphasize the importance of data-driven models and the identification of better biomarkers for characterizing symptom severity and closed-loop applications.

## Introduction

Parkinson’s Disease (PD) is associated with an excessive synchronization of beta oscillatory activity (13–30 Hz) in local field potentials (LFPs) recorded from the subthalamic nucleus (STN).^1^ Enhanced beta activity has been linked to bradykinesia and rigidity symptoms severity, providing a potential biomarker of disease state. Both the dopamine precursor levodopa^2^ and deep brain stimulation (DBS)^3^ disrupt enhanced beta activity, and the relative changes (OFF vs. ON medication/stimulation) in beta activity significantly correlate with clinical motor improvements.^4^ However, the relationship between PD OFF state beta activity and untreated clinical states has been largely inconsistent, questioning the mechanistic or epiphenomenal nature of beta oscillations. Indeed, some studies have found no significant correlation between beta activity at rest, and clinical scores in PD OFF state.^4^ Alternatively, several studies found a significant link with more complex metrics derived from beta,^5^ or have not taken into account the changes in clinical scores due to the surgical stun effect.^6^

**Fig. 1:**
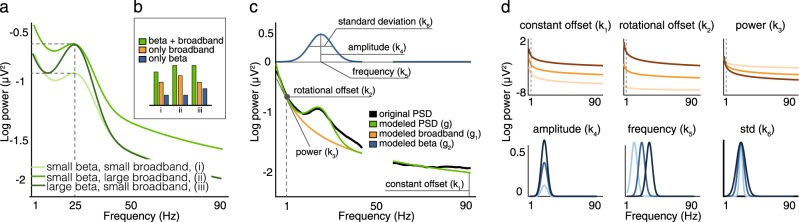
Effects of electrophysiological broadband activity on the estimation of beta activity. **a** Examples of simulated data representing how the 1/f broadband activity can lead to incorrect estimations of pathological beta rhythms. **b** Examples of how the estimation of beta changes when the broadband activity is small (i and iii) or large (ii). **c** Mathematical modeling of broadband activity and beta activity, together with the parameters estimated. The panel depicts the actual fitting from patient 5 (left hemisphere); the other model fits to individual patients are reported in Supplementary Figure 1. **d** Effect of changing one parameter on the broadband activity (top panel) and beta activity (bottom panel) functions; light color = small parameter value, neutral color = intermediate parameter value, dark color = large parameter value. See Supplementary Methods for details

We hypothesized that, in order to estimate an accurate beta biomarker for PD OFF state, it is needed to isolate beta activity from other ongoing physiological processes that could potentially confound the measurements. By default, measurements of STN beta oscillatory activity contain electrophysiological, broadband activity, which reflects random intrinsic electrical fluctuations within neuronal networks that display a 1/*f* spectral pattern. Recent works have shown that this broadband activity is a reliable neurophysiological indicator of the synaptic excitation/inhibition ratio,^7^ and have linked it with age and cognitive impairments.^8^ Given its physiological nature, we argue that broadband activity could impact and confound beta oscillatory activity, potentially explaining the large variability across studies (Fig. 1a, b).

Here we decompose the LFP power spectrum using a data-driven, automatic mathematical model as the sum of two independent neurophysiological elements: beta oscillatory activity modeled as a Gaussian function, and 1/f broadband activity corresponding to an exponential function (Fig. 1c, d and Supplementary Methods). We recorded 6 min of resting state STN LFPs bilaterally on thirteen PD patients (*N* = 26), and measured immediately afterwards their motor clinical scores (UPDRS part III motor examination, items 20 (tremor at rest), 22 (rigidity) and 23 (bradykinesia)). We evaluated the impact of broadband activity on the relationship between beta activity and motor symptoms, compared our model results with those obtained with actual data, and quantified the stability of both broadband and beta activity over time in order to determine the suitability of our approach for closed-loop deep brain stimulation.^9,10^

## Results

Goodness-of-fit (R^2^) between the modeled and actual power spectral densities (PSD) ranged between 0.95 and 0.99 (Supplementary Figure 1). There was a very strong correlation between the modeled beta activity and frequency and those found in the actual data (*r* = 0.97, *p* = 10^–7^, *r* = 0.62, *p* = 0.0006, respectively, see Supplementary Figure 2), confirming that our mathematical model accurately fitted the power spectrum and predicted the beta activity in an automated, data-driven manner (see Supplementary Methods and Results).

**Fig. 2:**
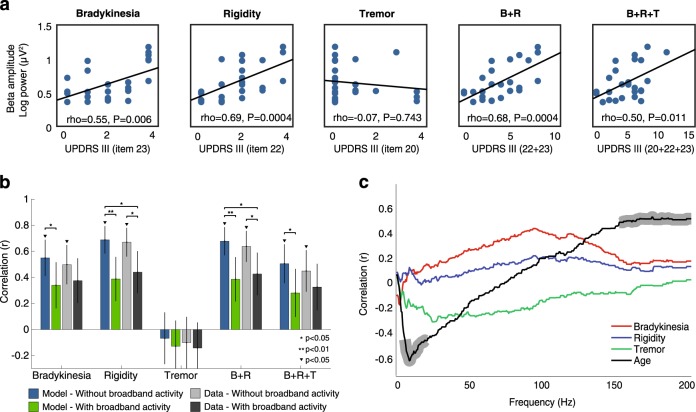
Isolated beta activity significantly improves prediction of clinical scores. **a** Spearman’s correlation coefficient between beta amplitude and motor symptoms (*N*=26; modeled PSD without broadband activity). **b** Comparisons of Spearman’s correlation coefficients between beta activity and motor symptoms, with and without broadband activity, and for both the modeled and the actual PSDs. Error bars denote standard deviation of the correlation coefficient (*p < 0.05, **p < 0.01, Hotelling’s *t* test for significant differences between correlations, FDR correction; ▼*p* < 0.05, test for significance for each correlation, FDR correction). **c** Spearman’s correlation between broadband activity and each motor symptom and age, at each frequency bin. Significant correlations are marked by shadowed regions (*p* < 0.05, FDR correction). B bradykinesia, R rigidity, T tremor

Removal of broadband activity from the estimation of beta activity yielded significantly higher correlations with clinical scores than the estimation of beta with broadband activity for bradykinesia, rigidity, bradykinesia plus rigidity, and all three clinical scores summed together (FDR corrected *p* < 0.05, Hotelling’s *t* test), but not for tremor (*p* = 0.32), Fig. 2a, b. While correlations between motor symptoms and beta activity without broadband activity were significant (Spearman’s correlation, FDR corrected *p* < 0.05), they were not for beta activity with broadband activity (FDR corrected p > 0.05 for all cases). Importantly, similar results were obtained when using the actual rather than the modeled power spectrum (see Fig. 2b): the correlations using actual PSDs were significant only when removing the broadband activity (FDR corrected *p* < 0.05 for Bradykinesia, Rigidity, *B* + *R* and *B* + *R* + *T*), but not when considering the broadband activity (*p* > 0.05). In this case, the improvement of correlation was significant for rigidity, and bradykinesia plus rigidity (FDR corrected *p* = 0.02 and *p* = 0.03, respectively).

We found no significant differences between the results using the model and those obtained using actual data (FDR corrected *p* > 0.2 for all cases). Despite this, the model approach still explained more variance (without broadband activity; Bradykinesia = 30%, Rigidity = 47%, Tremor = 0.45%, *B* + *R* = 46%, *B* + *R* + *T* = 26%) than in the actual data (without broadband activity; Bradykinesia = 25%, Rigidity = 45%, Tremor = 1%, *B* + *R* = 41%, *B* + *R* + *T* = 20%), indicating that the model explained more accurately the symptoms. Altogether, these results validate our modeling approach and suggest its benefits due to being automatic and data-driven.

Although we cannot rule out that the broadband activity might actually be a combination of device noise and true broadband activity, the same equipment was used for all the recordings performed. As such, potential impedance differences should average out at the group level. In addition, we found a significant negative correlation between age and broadband activity (Fig. 2c) for frequencies ranging between 7 and 21 Hz (FDR-corrected *p* < 0.05), and a positive correlation for frequencies ranging between 150 and 200 Hz (*p* < 0.05) in line with previous results,^8^ providing strong evidence against the non-physiological nature of the recorded broadband activity.

We also evaluated the variability of the broadband and beta activities at the sub-second level to assess the potential impact of broadband activity within an adaptive, closed-loop DBS scenario. To this end, we measured the standard deviation of beta and broadband activities over the recordings of one-second sliding windows with a 95% overlapping. Results showed that the variability throughout time of beta activity was significantly smaller than that of the broadband activity (variability across subjects for beta activity = 0.026 ± 0.028 and for broadband activity = 0.031 ± 0.027; Wilcoxon signed rank test; *p* < 10^–4^).

## Discussion

Despite advances in the understanding of the pathophysiology underlying PD, the causal role of beta activity remains unclear. This work presents further evidence of how resting oscillatory beta activity correlates with PD OFF states measured immediately after the recordings. Indeed, although Neumann et al. have found similar results, in their case, beta activity was correlated with pre-operative UPDRS III (OFF medication) measured between 1 to 12 weeks prior to the surgery.^6^ As such, this measure may not represent the actual clinical state during the recordings due to the long time period between measurements, and also due to the surgical stun effect. Other studies that successfully correlated beta biomarkers with PD OFF state have used more complex measures, such as entropy.^5^ Although these methods have implicitly tackled the variability induced by broadband activity, they have not explicitly addressed its impact when measuring beta as biomarkers. Here we showed how including broadband activity when estimating the beta biomarker led to non-significant correlations using modeled and actual data, while its removal led to a significant and more accurate estimation of the beta biomarker of symptom severity.

In this study, we evaluated for the first time the impact of the broadband activity on the estimation of beta activity. Our results prove that broadband electrophysiological activity confounded the measurements of beta oscillatory activity, support its physiological nature (significant correlation with age), and show its independence from other symptoms (lack of correlation with PD motor symptoms). It has been hypothesized that increases in broadband activity are a consequence of a pathological decoupling between population spiking activity and low-frequency oscillatory neural fields^11^ and is associated with an imbalance between synaptic excitation and inhibition.^7^

These results have a direct implication for closed-loop deep brain stimulation, where the stimulation is triggered only based on the appearance of a biomarker of the pathology.^9,10^ Our modeling approach potentially allows to extract a more reliable quantification of the proposed biomarker –pathological beta activity– as it encodes significantly better the severity of motor symptoms. In addition, we found that both beta oscillatory and broadband activity vary at the sub-second level extending previous works using computational models of broadband activity,^11^ and thus could impact the reliability of a closed-loop applications. As such, these results emphasize the benefits of modeling these neural components separately. Finally, our mathematical model is fully automatic, data-driven and personalized to patients and thus could lead to a more effective closed-loop therapy.^10^

## Methods

All 13 patients volunteered and gave written informed consent. Experimental protocol was approved by the local ethical committee (CER-VD, Commission Cantonale (VD) d’Ethique de la recherche sur l’être humain). Detailed methods are provided in the Supplementary Materials. This research has been previously published as a preprint.^12^

### Code availability

Data analyses were conducted in Matlab using scripts available from the corresponding author upon reasonable request.

## Electronic supplementary material

Supplementary Figures, Tables, Methods and Results

## Data Availability

The data that support the findings of this study are available from the corresponding author upon reasonable request.

## Electronic supplementary material

**Supplementary information** accompanies the paper on the *npj Parkinson’s Disease* website (10.1038/s41531-018-0068-y).
