# Supplementary material for: Differential contributions of subthalamic beta rhythms and 1/f broadband activity to motor symptoms in Parkinson’s disease
Source: NPJ Parkinsons Dis. 2018 Nov 5;4:32. doi: 10.1038/s41531-018-0068-y (PMC6218479; doi:10.1038/s41531-018-0068-y)
Supplement: Supplementary file 1 — Supplementary Figures, Tables, Methods and Results [file 41531_2018_68_MOESM1_ESM.pdf]

# Supplementary Materials

## Differential contributions of subthalamic beta rhythms and 1/f broadband activity to motor symptoms in Parkinson's disease

Stephanie Martin[a,b†], Iñaki Iturrate[a†*], Ricardo Chavarriaga[a], Robert Leeb[a], Aleksander Sobolewski[a], Andrew M. Li[a,c], Julien Zaldivar[d,e], Iulia Peciu-Florianu[d], Etienne Pralong[d], Mayte Castro-Jiménez[d], David Benninger[d], François Vingerhoets[d], Robert T. Knight[b,f], Jocelyne Bloch[d†], José del R. Millán[a†]

[†]equal contribution

[a] Defitech Chair in Brain-Machine Interface, Center for Neuroprosthetics, Ecole Polytechnique Fédérale de Lausanne, Switzerland

[b] Helen Wills Neuroscience Institute, University of California, Berkeley, CA, USA

[c] Li Ka Shing Faculty of Medicine, University of Hong Kong, Hong Kong, P.R. China

[d] Department of Clinical Neurosciences (Neurology and Neurosurgery), University Hospital of Vaud (CHUV), Lausanne, Switzerland

[e]Service de Neurochirurgie, Hôpital de Sion, Hôpital du Valais, Switzerland.

[f] Department of Psychology, University of California, Berkeley, CA, USA

***Corresponding author:** Iñaki Iturrate, CNBI, Center for Neuroprosthetics, EPFL, Ch. des Mines 9, 1202 Genève, Switzerland, inaki.iturrate@epfl.ch

### Acronyms

| | |
|---|---|
| LFP | Local field potentials |
| STN | Subthalamic nucleus |
| DBS | Deep brain stimulation |
| UPDRS | Unified Parkinson's Disease rating scale |
| CT | Computerized tomography |
| MRI | Magnetic resonance imaging |
| PSD | Power spectral density |
| FDR | False discovery rate |

# Supplementary methods

## Subjects and experimental protocol

LFPs were recorded between 36 and 48 hours after STN DBS bilateral implantation in thirteen subjects using a 16-channel amplifier (gUsbAmp, g.tec, Austria) and sampled at 512 Hz. All patients volunteered and gave written informed consent. Experimental protocol was approved by the local ethical committee (CER-VD, Commission Cantonale (VD) d'Ethique de la recherche sur l'être humain). Clinical characteristics of each patient are presented in Supplementary Table 1.

Patients sat comfortably in a semi-recumbent position in their hospital bed. LFPs were recorded bilaterally during six minutes in a resting state condition. During the recording, patients were asked to restrain from performing any movements nor speaking, and maintaining their eyes open. Prior to recording, the stimulation device was turned off for at least ten minutes. Patients were instructed to relax and keep their eyes open during the recording. Patients were clinically assessed at the end of the recording by independent neurologists using the Unified Parkinson's Disease Rating Scale part III Motor Examination (UPDRS III) sub-scores for bradykinesia (item 23), rigidity (item 22) and resting tremor symptoms (item 20) for both upper limbs.

## Surgical procedure

Long-acting dopaminergic medication was withdrawn 48 hours prior and short-acting medication was withdrawn 12-20 hours prior to off-medication pre-operative testing and to the DBS lead implantation procedure, always performed by the same surgeon. The awake surgical procedure stereotactically implanted a 3389 Medtronic electrode in the motor part of the STN. Direct surgical planning was performed with the Medtronic stealth station, based on a CT scan obtained with the CRW stereotactic frame, and fused with two 3 Tesla MRI sequences (MPRAGE with Gadolinium and space T2), obtained prior to surgery. The precise trajectory of the implanted electrode was simulated and its exact coordinates were calculated in relation with the position of the stereotactic frame. During the surgery, a cannula was implanted towards the STN following the calculation of the trajectory and remained in place until the end of the surgery. Through this cannula, a microelectrode was lowered toward the target in the STN and the border of the motor STN was visualized as the transition from slow spiking neurons to fast spiking neurons. After a first phase

of microrecording, macrostimulation was performed under neurological clinical assessment; improvement of rigidity was expected in case of correct placement. Adjustment of the electrode placement through a second track was eventually performed to optimize the clinical response to stimulation. The electrode location was checked during the surgery with the O-Arm (Medtronic). An externalized extension cable was connected to the distal part of the electrode and tunneled posterior to the skin incision to record the brain activity for a period of 3 days. Internalization of the electrode and connection of the electrode to an internal stimulator were performed at day 4 under general anesthesia. Once the definitive electrode was placed through the same cannula to the definitive target, a second CT scan was obtained after having implanted the definitive electrode. Finally, the electrode was fixed with cement on the skull.

In order to assess the efficacy of stimulation, we calculated the clinical score during OFF- versus ON-stimulation. Results showed that the clinical scores were significantly better during ON-stimulation than during OFF-stimulation across patients and hemispheres ($mean_{ON}$= 2.6±2.2, $mean_{OFF}$= 5.2±3.9; p=0.0001, signed rank test). The improvement in clinical score from OFF- to ON-stimulation was calculated as follows:

$$improvement(\%) = 100 \cdot \frac{UPDRS_{OFF} - UPDRS_{ON}}{UPDRS_{OFF}}$$

The average improvement across patients and hemisphere was 43±7%, similar to those obtained by previous works[1].

## Power spectrum modeling

Six minutes of bipolar LFP activity were derived from two contacts, selected based on the electrodes chosen by the neurosurgeon for DBS stimulation. LFPs were first high-pass filtered at 1 Hz using a second order Butterworth filter. Artifact were removed using an automatic threshold approach (data with activity higher than 50μV in absolute value), followed by visual inspection and rejection of contaminated portions of data. Those windows with abnormal activations (higher than 50 μV for the automatic approach; or marked as noisy by visual inspection) were eliminated from further analyses. Altogether, the recordings were very clean in all participants, but one

(Patient 10). The amount of data rejected for this patient was almost 20%, whereas it was <1% for all other patients. Power spectral densities (PSD) were then calculated using a Short-Time Fourier Transform, with a 0.25s Hamming window and 60% overlap, and finally log powered.

We propose a model that describes the LFP spectrum and allows the extraction of two components of the PSD: the broadband activity and the beta oscillatory activity. Similar approaches have been used to model the PSDs in EEG[2,3]. The different components of the model are illustrated in Figure 1c (main text), in which the LFP PSDs are modeled as:

$$g(f) = g_1(f) + g_2(f) = k_1 + \frac{k_2}{f^{k_3}} + k_4 \cdot e^{\frac{(f-k_5)^2}{2{k_6}^2}}$$

where $g(f)$ is the modeled power spectrum at frequency $f$.

The function $g_1(f)$ models the broadband activity function at frequency $f$ with a power law function; $k_1$, $k_2$, $k_3$. Parameter $k_1$ determines the constant offset of the PSD. Parameter $k_3$ represents the speed of decay of the 1/f function, and can be visualized as the slope at f=1Hz. Finally, parameter $k_2$ allows to change the rotation axis of the $1/f^{k_3}$ function. If $k_2$=1, the rotation axis of $1/f^{k_3}$ will always occur at 1 $\mu V^2$ at f=1Hz, no matter the value of $k_3$. The speed of decay $k_3$ defines how the PSD rotates around this axis. Parameter $k_3$ allows to shift the rotation axis to a different value than 1 $\mu V^2$ at f=1Hz, which may allow to model more realistically neurophysiological phenomena[3].

The function $g_2(f)$ models the beta activity function at frequency $f$ with a Gaussian probability density function, with amplitude $k_4$, mean frequency $k_5$, and standard deviation $k_6$. Fig. 1d (main text) exemplifies the impact of changing each of the parameters on the modeled broadband (top row) or beta activity (bottom row), respectively. $k_{1-6}$ parameters were estimated from the entire 6-min recording by an interior-point optimization algorithm, with the objective function defined as the root mean square error between the modeled and actual PSDs:

$$\min_{\boldsymbol{k}\in\mathbb{R}^6}\sum_{f}(PSD(f)-g(f,\boldsymbol{k}))^2$$

where frequencies $f$ ranged between 8-90 Hz, except for frequencies between 48-52Hz which were not taken into account for the modeling due to the use of a notch filter to remove power line noise. We have ignored frequencies below 8Hz in order to avoid low frequency artifacts[1] and tremor-associated activity[4]. Lower and upper bounds on the design variables were defined based on a priori knowledge and the shape of typical power spectral densities. Initial values of parameters were set to the central value over the range defined by the lower and upper bounds (see Supplementary Table 2).

Figure 1 (main text) exemplifies how the broadband activity level might affect the quantification of beta oscillatory activity. The light green curve shows a small beta amplitude and a low level of broadband activity evaluated at the peak amplitude (25 Hz). Alternatively, the neutral green curve has the same beta amplitude as the light green curve, but the level of broadband activity is larger, resulting in an overall larger total amplitude. Finally, the dark green curve has the same overall amplitude as the neutral green curve, but the beta amplitude is actually much larger. These examples emphasize the need to model these physiological processes independently.

Although, we cannot rule out that the broadband activity might actually be a combination of device noise and true broadband activity, the same equipment was used for all the recordings performed. As such, potential impedance differences should average out at the group level (patients and hemispheres), and results obtain should underlie physiological processes.

Emerging evidence has also shown the existence of two distinct beta sub-bands carrying pathological information[5]: the low-beta (13–20 Hz) and the high-beta (20–35 Hz) bands. As such, we also evaluated the feasibility to model the PSDs with two beta bumps. To this end, the PSD was modeled as the sum of two Gaussian function and a power law function, and therefore estimated nine parameters instead of six parameters in the case of a single beta bump.

## Statistical analysis

To validate our model, we first evaluated the goodness-of-fit between the modeled and actual PSDs using the coefficient of determination ($R^2$). We then quantified the relationship between the model parameters $k_{1-6}$ and the clinical scores using Spearman's correlation coefficient (rho), pooling patients and hemispheres (N=26). The significance level was computed using exact permutation tests and corrected for multiple comparisons using false discovery rate (FDR)[6].

We then compared differences in correlation with clinical scores, when the beta oscillatory activity was estimated with and without broadband activity. The estimated beta activity without broadband corresponded to the amplitude of the Gaussian function ($k_4$). On the other hand, the beta activity estimated with the broadband activity corresponded to the value of the modeled PSDs evaluated at the frequency estimated with our model, $g(k_5)$, which equaled the sum of both, the amplitude of the Gaussian function ($k_4$) and the value of the modeled broadband activity function evaluated at the center frequency of the Gaussian function, $g_1(k_5)$. We assessed for significant differences between correlation values with and without broadband activity using FDR corrected Hotelling's t-statistics. This statistical test accounts for the dependence of the two correlations on the same group, i.e. both correlations are relative to the same motor symptoms.

To validate our approach, we performed the same analysis using the actual PSDs, and compared the results with those obtained using the modeled PSDs. For this, the beta peak in the actual PSDs for each patient and hemisphere was determined by an external expert blinded to the study. Then, we evaluated the correlation of the beta amplitude in the actual PSDs with motor symptoms, with and without the modeled broadband activity.

Finally, we investigated the relationship between the broadband activity and motor symptoms and age, as previous works have shown that age correlates with broadband activity within specific frequencies[7]. As such, we evaluated for each patient and hemisphere (N=26) the broadband activity function (function $g_1$, see Figure 1c in the main text) within the frequencies $f \in [0,200]$ Hz, based on the parameters estimated from the model. Then, for each evaluated frequency $f$, we correlated $g_1(f)$ with the symptoms (Spearman's correlation) and obtained the associated p-value of the correlation. Once all the correlations were performed, we corrected the p-values for multiple

comparisons using the false discovery rate method (FDR)[6]. For the correlation of the broadband activity function with age, we associated the same age to both hemispheres for a given patient.

## Code availability

Data analyses were conducted in Matlab using scripts available from the corresponding author upon reasonable request.

## Data availability

The data that support the findings of this study are available from the corresponding author upon reasonable request.

# Supplementary results

## Modeling of the power spectrum

Goodness-of-fit ($R^2$) between the modeled and actual PSDs ranged between 0.95-0.99, and thus our mathematical model accurately fitted the power spectrum (see Supplementary Figure 1). While Parkinson's disease has often been associated with two beta frequencies (low beta (8-20Hz) and high beta (21-35Hz))[5], our rationale for performing the analysis on a single bump was two-fold: first, there were no significant differences across conditions in the adjusted $R^2$-which takes into account the number of parameters as a regularization method (mean $R^2_{oneBeta}$=0.98 and mean $R^2_{twoBeta}$=0.98, p = 0.16; signed rank test); and second, we found no significant differences across conditions in the Akaike information criteria, and it was lower on average for the case of one beta bump (mean $AIC_{oneBeta}$= 496 and mean $AIC_{twoBeta}$ = 525; p = 0.43; signed rank test). These results suggest that power spectral densities can be modeled using customizable parameters depending on the needs. Despite this, and to avoid an overfitting effect when modeling two bumps, we limited our investigation to modeling beta activity using only a single beta bump.

## Correlation between model parameters and motor symptoms

In order to identify how the LFP power relates to motor symptoms, we investigated the relationship between the different parameters of the modeled power spectrum and the UPDRS III clinical scores. The fitted amplitude of the beta activity ($k_4$) correlated with both bradykinesia (rho=0.55, $p$=0.006) and rigidity (rho=0.69, $p$=0.000), but not with tremor (rho=-0.07, $p$=0.743; Figure 2 main text). In addition, the beta amplitude correlated with bradykinesia plus rigidity (rho=0.68, $p$=0.000) and all three clinical scores summed together (rho=0.50, $p$=0.011). No other parameters presented significant correlations ($p$>0.1; Supplementary Table 3). These findings agree with previous electrophysiological studies showing that pathological beta activity was associated with bradykinesia and rigidity, but not tremor[8].

Similar to the results obtained with our model, the correlations between motor symptoms and beta amplitude using actual PSDs were significant only when removing the broadband activity (FDR corrected p<0.05 for Bradykinesia, Rigidity, B+R and B+R+T); but they were not significant when

the broadband activity was considered ($p>0.05$). In addition, the increase in correlation between with and without broadband activity was significant for Rigidity (Hotelling's t-test, FDR corrected $p=0.02$) and B+R (FDR corrected $p=0.03$).

We found no significant differences between the results using the model and those obtained using real data (FDR corrected $p>0.2$ for all motor symptoms). Despite this, the model approach (without broadband) still explained more variance (Bradykinesia=30%, Rigidity=47%, Tremor=0.45%, B+R=46%, B+R+T=26%) than the real data (without broadband; Bradykinesia=25%, Rigidity=45%, Tremor=1%, B+R=41%, B+R+T=20%).

To further evaluate the validity of our model, we compared the beta amplitude and beta frequency estimated by the model with those obtained using the actual PSDs. Results showed a very strong correlation between the beta power in the real data and the modeled beta peak (Supplementary Figure 2; $r=0.97$; $p=10^{-7}$). Similarly, the correlation between the modeled beta frequency and the real beta frequency was also very high ($r=0.62$, $p=0.0006$), further highlighting the accuracy of the model.

# Supplementary figures

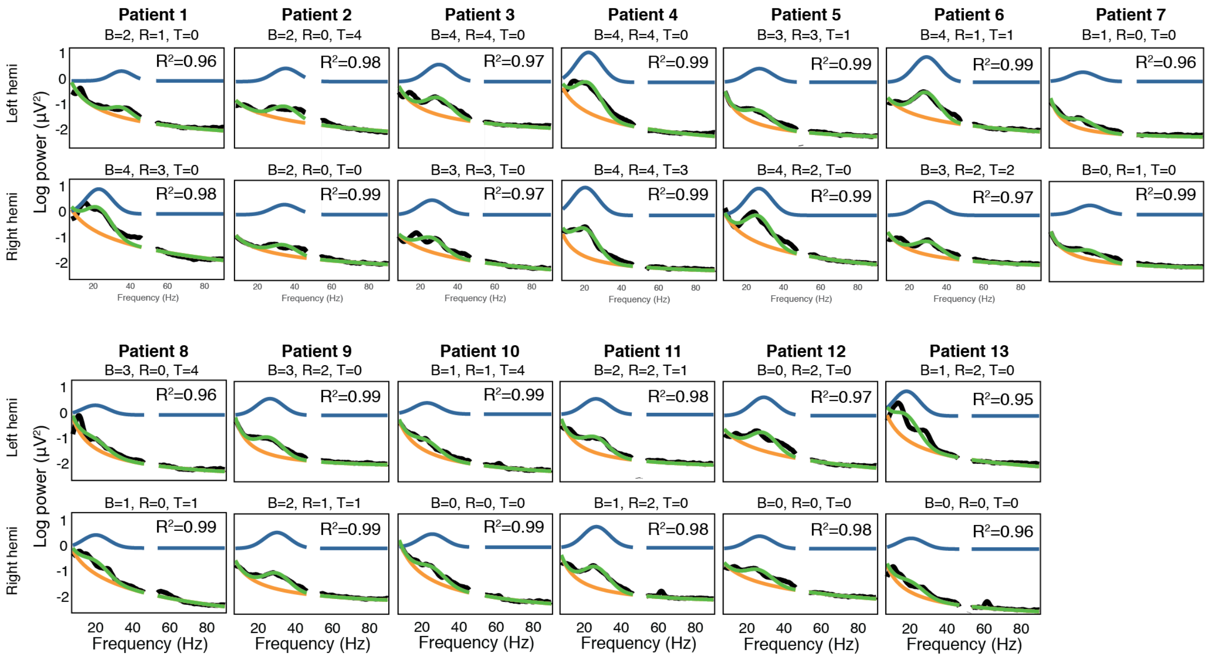


**Supplementary Figure 1. Model fits to individual datasets.** Model fits (orange = broadband activity, blue = beta activity and green = sum of broadband and beta activity) for each patient and hemisphere, together with the actual power spectral densities (black lines). For each subject and hemisphere, the bradykinesia, rigidity and tremor scores are also reported, as well as the individual $R^2$ goodness of fit. Note that the fitting was not performed (nor shown) within the 48-52Hz due to the use of a notch filter.

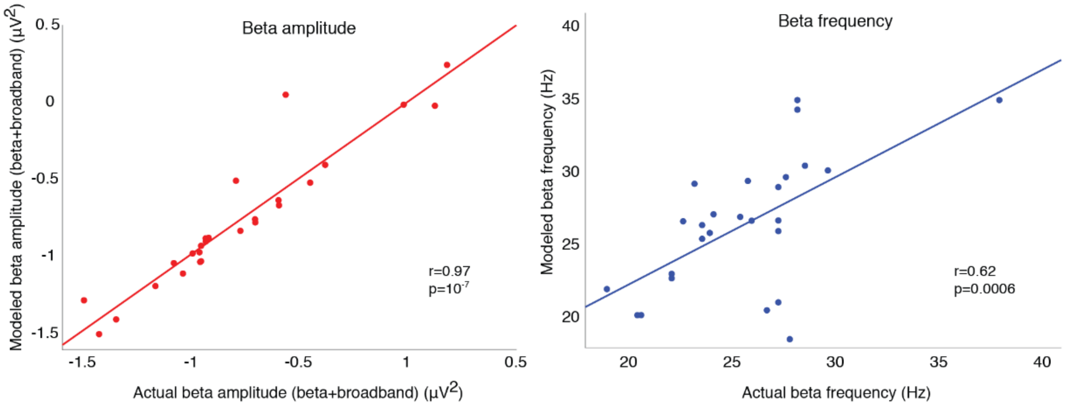


**Supplementary Figure 2.** Comparison between the modeled and actual data for the beta amplitude (left panel) and the beta frequency (right panel) across patients and hemispheres.

# Supplementary tables

***Supplementary Table 1: Clinical details of patients***

| Patient | *Disease duration (years)* | *Age (years)* | *Gender (M/F)* | *UPDRS III (23+22+20)* | | *Bradykinesia (UPDRS III - 23)* | | *Rigidity (UPDRS III - 22)* | | *Tremor (UPDRS III - 20)* | | Drugs prior to surgery (LED mg)[2] |
|---|---|---|---|---|---|---|---|---|---|---|---|---|
| | | | | *left* | *right* | *left* | *right* | *left* | *right* | *left* | *right* | |
| **P01** | 15 | 60 | M | 3 | 7 | 2 | 4 | 1 | 3 | *0* | *0* | 702 |
| **P02** | 11 | 81 | F | 4 | 4 | 2 | 2 | 0 | 0 | *4* | *0* | 950 |
| **P03** | 7 | 62 | M | 8 | 6 | 4 | 3 | 4 | 3 | *0* | *0* | 1143 |
| **P04** | 15 | 68 | M | 8 | 11 | 4 | 4 | 4 | 4 | *0* | *3* | 1250 |
| **P05** | 22 | 56 | M | 7 | 6 | 3 | 4 | 3 | 2 | *1* | *0* | 613 |
| **P06** | 10 | 70 | M | 6 | 7 | 4 | 3 | 1 | 2 | *1* | *2* | 1009 |
| **P07** | 11 | 77 | M | 1 | 1 | 1 | 0 | 0 | 1 | *0* | *0* | 1052 |
| **P08** | 14 | 65 | F | 7 | 2 | 3 | 1 | 0 | 0 | *4* | *1* | 1144 |
| **P09** | 16 | 74 | M | 5 | 4 | 3 | 2 | 2 | 1 | *0* | *1* | 911 |
| **P10** | 6 | 62 | F | 6 | 0 | 1 | 0 | 1 | 0 | *4* | *0* | 1512 |
| **P11** | 15 | 70 | M | 5 | 3 | 2 | 1 | 2 | 2 | *1* | *0* | 994 |
| **P12** | 18 | 67 | M | 2 | 0 | 0 | 0 | 2 | 0 | *0* | *0* | 1384 |
| **P13** | 11 | 66 | M | 3 | 4 | 1 | 2 | 2 | 2 | *0* | *0* | 1324 |
| **µ±SEM** | 13.2±4.4 | 67.5±6.8 | *N/A* | *5.0*±2.3 | *4.2*±3.2 | *2.3*±1.3 | *2.0*±1.5 | 1.7±1.4 | 1.5±1.3 | 1.2±1.7 | 0.5±1.0 | 1076.0±257.4 |

[1] Sum of UPDRS III items 20, 22, 23 and both hemispheres, during OFF-medication and OFF-stimulation conditions.
[2] Levodopa Equivalent Dose.

### *Supplementary Table 2: Model parameters*

| Parameter | Lower bound | Upper bound | Initial value |
|---|---|---|---|
| $\boldsymbol{k_1}$, offset | -10 | 10 | 0 |
| $\boldsymbol{k_2}$, rotational offset | 0.01 | 20 | 10 |
| $\boldsymbol{k_3}$, power | 0.01 | 2 | 1 |
| $\boldsymbol{k_4}$, amplitude | 0 | max(PSD) | max(PSD) |
| $\boldsymbol{k_5}$, mean | 8 | 35 | 21.5 |
| $\boldsymbol{k_6}$, standard deviation | 2 | 7 | 4.5 |

*Arbitrary units*

### *Supplementary Table 3: Relationship between model parameters and motor symptoms*

| Parameter | mean±std | corr(B) | corr(R) | corr(T) | corr(B+R) | corr(B+R+T) |
|---|---|---|---|---|---|---|
| $\boldsymbol{k_1}$, offset | -4.33±2.93 | -0.04 | 0.14 | 0.11 | 0.08 | 0.04 |
| $\boldsymbol{k_2}$, rotational offset | 9.80±3.61 | -0.25 | 0.01 | -0.01 | -0.15 | -0.17 |
| $\boldsymbol{k_3}$, power | 0.50±0.33 | -0.08 | 0.07 | 0.08 | 0.02 | 0.04 |
| $\boldsymbol{k_4}$, amplitude | 0.65±0.25 | **0.55*** | **0.69**** | -0.07 | **0.68**** | **0.50*** |
| $\boldsymbol{k_5}$, mean | 26.49±4.58 | -0.10 | -0.21 | -0.04 | -0.17 | -0.18 |
| $\boldsymbol{k_6}$, standard deviation | 6.96±0.22 | 0.25 | 0.32 | 0.01 | 0.32 | 0.25 |

**P*<0.05, ***P*<0.01, FDR correction

B: Bradykinesia; R: Rigidity; T: Tremor

## Supplementary references


1. Kühn, A.A., Tsui A., Aziz T., Ray, N. Brücken C., Kupsch A., Schneider G.H & Brown, P. Pathological synchronisation in the subthalamic nucleus of patients with Parkinson's disease relates to both bradykinesia and rigidity. *Experimental neurology* **215**, 380-387 (2009).

2. Blankertz, B., Sannelli, C., Halder, S., Hammer, E.M, Kübler, A., Müller, K.R., Curio, G. & Dickhaus, T. Neurophysiological predictor of SMR-based BCI performance. *NeuroImage*. **51**, 1303–1309 (2010).

3. Gao, R. Interpreting the electrophysiological power spectrum. *Journal of neurophysiology* **115**, 628-630 (2015).

4. Reck, C., Florin, E., Wojtecki, L., Krause, H., Groiss, S., Voges, J., Maarouf, M., Sturm, V., Schnitzler, A. & Timmermann, L. Characterisation of tremor-associated local field potentials in the subthalamic nucleus in Parkinson's disease. *European journal of neuroscience* **29**, 599-612 (2009).

5. Priori, A., Foffani, G., Pesenti, A., Tamma, F., Bianchi, A.M., Pellegrini, M., Locatelli, M., Moxon, K.A. & Villani, R.M. Rhythm-specific pharmacological modulation of subthalamic activity in Parkinson's disease. *Experimental neurology* **189**, 369-379 (2004).

6. Benjamini, Y. & Hochberg, Y. Controlling the false discovery rate: a practical and powerful approach to multiple testing. *Journal of the royal statistical society. Series B (Methodological)* **57**, 289-300 (1995).

7. Voytek, B., Kramer, A.M., Case, J., Lepage, K.Q., Tempesta, Z.R., Knight, R.T. & Gazzaley, A. Age-related changes in 1/f neural electrophysiological noise. *Journal of Neuroscience* **35**, 13257-13265 (2015).

8. Ray, N.J., Jenkinson, N., Wang, S., Holland, P., Brittain, J.S., Joint, C., Stein, J.F. & Aziz, T. Local field potential beta activity in the subthalamic nucleus of patients with Parkinson's disease is associated with improvements in bradykinesia after dopamine and deep brain stimulation. *Experimental neurology* **213**, 108-113 (2008).
